# Elevated Macrophage Migration Inhibitory Factor 1 Is Associated with Left and Right Ventricular Systolic Dysfunction in Heart Failure with Reduced Ejection Fraction

**DOI:** 10.3390/biomedicines13051087

**Published:** 2025-04-30

**Authors:** Timea Magdolna Szabo, Mihály Vass, Márta Germán-Salló, Attila Frigy, Előd Ernő Nagy

**Affiliations:** 1 Department of Biochemistry and Environmental Chemistry, George Emil Palade University of Medicine, 540142 Targu Mures, Romania; timea.szabo@umfst.ro (T.M.S.); elod.nagy@umfst.ro (E.E.N.); 2 Department of Cardiology, Clinical County Hospital Mures, 540103 Targu Mures, Romania; 3 Department of Anesthesiology, Emergency Institute for Cardiovascular Disease and Heart Transplantation, 540136 Targu Mures, Romania; mihalyvass23@gmail.com; 4 Department of Internal Medicine III, George Emil Palade University of Medicine, 540142 Targu Mures, Romania; marta.german-sallo@umfst.ro; 5 Department of Internal Medicine IV, George Emil Palade University of Medicine, Pharmacy, Science and Technology of Targu Mures, 540142 Targu Mures, Romania; 6 Laboratory of Medical Analysis, Clinical County Hospital Mures, 540394 Targu Mures, Romania

**Keywords:** heart failure, heart failure with reduced ejection fraction, macrophage migration inhibitory factor 1, systemic inflammation

## Abstract

**Background/Objectives**: Low-grade systemic inflammation, characteristic of heart failure (HF), is a nonspecific inflammatory syndrome that affects the entire body. Macrophage migration inhibitory factor 1 (MIF-1) is a pro-inflammatory cytokine, a key mediator of the innate immune response, and may serve as a potential biomarker of monocyte homing and activation in HF with reduced and mildly reduced ejection fraction (HFrEF, HFmrEF). **Methods**: We evaluated 70 hemodynamically stable patients with left ventricular EF (LVEF) < 50% by means of echocardiography and blood sampling. **Results**: We report significant correlations between MIF-1, LVEF (r = −0.33, *p* = 0.005), LV global longitudinal strain (LVGLS, r = 0.41, *p* = 0.0004), and tricuspid annular plane systolic excursion (TAPSE, r = −0.37, *p* = 0.001). MIF-1 levels in HFrEF patients were relatively higher, but not significantly different from those observed in HFmrEF. MIF-1 showed significant associations with TAPSE to systolic pulmonary artery pressure ratio (TAPSE/sPAP, *p* < 0.0001). Also, patients with TAPSE/sPAP < 0.40 mm/mmHg had significantly higher levels of MIF-1 (*p* = 0.009). Moreover, ischemic cardiomyopathy (ICM) was more frequent in patients with MIF-1 concentrations above 520 pg/mL (57.1% MIF-1^hi^ vs. 28.6% MIF-1^lo^, *p* = 0.029). In terms of congestion, MIF-1 showed significant associations with the presence of peripheral edema (*p* = 0.007), but none was found with self-reported dyspnea (*p* = 0.307) and New York Heart Association (NYHA) class (*p* = 0.486). Also, no relationship was reported with N-terminal pro-B-type natriuretic peptide concentrations (NT-proBNP, r = 0.14, *p* = 0.263). However, the six-minute walk distance was greater in individuals in the MIF-1^lo^ group when compared to those in the MIF-1^hi^ group (404.0 ± 127.4 vs. 324.8 ± 124.1 m, *p* = 0.010). **Conclusions**: Beyond identifying inflammatory biomarkers related to disease severity, linking MIF-1 to various pathophysiological mechanisms may highlight the active involvement of the monocyte-macrophage system in HF. This system holds notable significance in congestion-related conditions, acting as a major source of reactive oxygen species that perpetuate inflammation.

## 1. Introduction

Heart failure (HF) is a condition of particular interest among physicians worldwide, due to its rapidly progressive course and increasing demand for long-term care [1]. Also, HF remains a major cause of cardiovascular death and disability [2]. Inflammation and HF are strongly connected, as inflammation proved to be a prognostic risk factor and a key mediator of disease progression in HF [3]. A large number of cytokines were described in HF over the last three decades since tumor necrosis factor α (TNF-α) was first linked to cardiac cachexia [4]. Furthermore, the active participation of the monocyte-macrophage system in multiple degenerative and inflammatory conditions suggests a possible role played by the macrophage migration inhibitory factor (MIF) in the complex pathophysiology of HF [3,5].

MIF-1 is an atypical chemokine which acts as an essential mediator of the innate immune response. MIF is expressed as a 12.5 kDa protein which is biologically active as a homotrimer [6,7,8]. MIF-2, also known as D-dopachrome tautomerase (D-DT), contains 117 amino acid residues, and has been shown to be a structural and functional homolog of MIF-1 [9]. Moreover, both mediators exhibit enzyme activity [6,7,8,9]. MIF was first described nearly six decades ago by Bloom et al. in an experimental model as a molecule that inhibits macrophage migration and is secreted by lymphocytes [10].

MIF shows ubiquitous and constitutive expression in nearly all mammalian cells (both immune and non-immune), and is stored in intracellular vesicles. MIF plays an active role in numerous biological processes, mediated through autocrine, paracrine, and endocrine signaling, by binding to the CD74, CXCR2, CXCR4, and CXCR7 receptors, which mediate upstream intracellular signaling even through receptor complex formation. Not only it contributes to pathogen clearance in infectious diseases, but it also amplifies inflammatory response, promotes cell proliferation, and counteracts the anti-inflammatory and immunosuppressive activity of endogenous glucocorticoids [6,7,8]. Intracellular MIF showed cardioprotective effects in the early stages of myocardial ischemia/reperfusion (I/R) injury through receptor-driven modulation of the CD74/AMPK signaling cascade and the attenuation of oxidative stress [11,12,13]. The same benefits were achieved by the pharmacologic augmentation of MIF using MIF20 (MIF agonist) in the senescent myocardium [14]. However, elevated serum concentrations have been associated with higher mortality rates among critically ill patients [15] and in HF regardless of EF [16]. Post-translational modifications observed ex vivo alter MIF activity [9], which may explain its dichotomous role.

MIF is rapidly released from cells in response to infectious, inflammatory, metabolic, and environmental stimuli, exerting pro-inflammatory and immunomodulatory effects. MIF stimulates the release of other cytokines and mediators, TNF-α, interleukin 1β (IL-1β), IL-6, IL-8, nitric oxide, and cyclooxygenase. Additionally, through its neutralizing impact on corticosteroids, mitogen-activated protein kinase phosphatase 1 cannot inhibit TNF-α messenger ribonucleic acid (mRNA) translation, thus further promoting inflammation [8]. The pharmacological targeting of MIF signaling pathways and receptors using small synthetic molecules (nanobodies), antibodies, or receptor-targeted peptides, is of particular interest in cancer treatment, as it counteracts the pro-proliferative effects of MIF and D-DT. The proline-1 residue is essential for MIF to exert its enzymatic activity, which supports the rationale for pharmacologically targeting the active site of MIF to inhibit the cytokine [17,18]. Studies aiming to neutralize MIF used mainly in vitro or in vivo murine models of skin cancer (multiple melanoma), gastric cancer, and endotoxin shock [19,20,21,22]. Therapy with imalumab, a humanized monoclonal antibody targeting the oxidized form of MIF, resulted in stable disease progression in 26% of patients with metastatic colorectal cancer [23]. Milatuzumab, an anti-CD74 humanized monoclonal antibody, is currently approved for the treatment of refractory multiple myeloma [24], while the CD74 receptor antagonist, RTL1000, is under investigation for multiple sclerosis [25]. Ibudilast not only possesses anti-inflammatory and neuroprotective properties as a non-selective phosphodiesterase inhibitor and toll-like receptor 4 antagonist, but also suppresses glial cell activation. This allosteric modulator of MIF is approved in Japan for the treatment of bronchial asthma and stroke. Due to its pharmacokinetic properties that allow it to cross the blood-brain barrier, ibudilast is currently being evaluated as a therapeutic option in glioblastoma [17,26].

The widespread expression of MIF underscores its potential as a biomarker for predicting clinical disease progression by reflecting the systemic impact of HF. Furthermore, MIF-1 antagonization may offer therapeutic benefits in HF, considering the pivotal role of systemic inflammation in its pathophysiology.

## 2. Materials and Methods

### 2.1. Research Design and Method

Seventy patients with HF and left ventricular ejection fraction (LVEF) < 50% were enrolled from the Cardiology Department of the Mures County Clinical Hospital, Targu Mures, Romania. All participants were hemodynamically stable at the time of examination. A brief overview of the current study protocol was provided in a previous publication [5]. Patients were excluded from the study if they presented with signs or symptoms of infection, or had a documented diagnosis of cancer, autoimmune disease, liver disease, or significantly impaired kidney function—defined as an estimated glomerular filtration rate (eGFR) below 20 mL/min/1.73 m^2^, calculated using the Chronic Kidney Disease Epidemiology Collaboration (CKD-EPI) equation. Demographic, clinical (including body mass index (BMI)), laboratory, and ultrasound (both heart and lung) data were systematically collected for all participants. Functional capacity and risk stratification were further evaluated using the six-minute walk test (6MWT) distance. Health-related quality of life was also assessed by applying the Minnesota Living with Heart Failure Questionnaire (MLHFQ).

The study adheres to the Declaration of Helsinki. Institutional ethics committees approved the study protocol (7716/2 July 2021, Mures County Clinical Hospital; 2281/13 April 2023, “George Emil Palade” University of Medicine, Pharmacy, Science, and Technology of Targu Mures), and all participants signed an informed consent prior to recruitment.

### 2.2. Heart and Lung Ultrasound

Echocardiography was performed using a Philips Epiq7 device (Philips Ultrasound, Inc., Bothell, WA, USA) and a Philips X5-1 xMATRIX probe (1–5 MHz). LV systolic function was assessed by means of EF and global longitudinal strain (GLS). LVEF was measured using the modified Simpson’s biplane rule, while LVGLS was calculated by averaging data acquired from the three standard apical views. Tricuspid annular plane systolic excursion (TAPSE) to systolic pulmonary artery pressure (sPAP) ratio was also determined, and TAPSE/sPAP < 0.40 mm/mmHg was defined as a negative prognostic marker [27].

Lung ultrasound (LUS) was also performed by using the same cardiac ultrasound transducer in order to search for residual pulmonary congestion. All patients were evaluated in a semi-seated position while the anterior and lateral chest walls were scanned for B-lines. The eight-zone (four on each hemithorax) LUS protocol was chosen, establishing the following threshold: normal with 0–2 B-lines/zone, and abnormal with ≥3 B-lines/zone [28,29].

### 2.3. MIF-1 and IL-6 ELISA

Blood was separated by centrifugation (3000 rpm for 10 min), then the serum was transferred into 1.5 mL Eppendorf tubes and stored at −50 °C. Circulating levels of MIF-1 (Human MIF DuoSet ELISA, RD-DY289) and IL-6 (Human IL-6 DuoSet ELISA, RD-DY206) were measured using commercially available immunoassay kits manufactured by R&D Systems (Minneapolis, MN, USA). Ancillary reagents were also used (DuoSet ELISA Ancillary Reagent Kit 2, RD-DY008; R&D Systems, Minneapolis, MN, USA). Results were read on a Personal Lab ELISA automated analyzer (Adaltis, Milano, Italy).

Since currently there is no validated reference range for MIF-1 in HF with reduced EF (HFrEF) and HF with mildly reduced EF (HFmrEF), we divided our patients into two groups, labeled as MIF-1^lo^ and MIF-1^hi^, using the median as a cut-off value—520 pg/mL. This allowed the dichotomous categorization of data and the comparison of groups.

### 2.4. Laboratory Analyses

Biochemical analysis (total, low-density lipoprotein (LDL) and high-density lipoprotein (HDL) cholesterol, serum triglycerides, uric acid, creatinine, albumin, serum iron, ferritin, C-reactive protein (CRP), glycemia, gamma-glutamyl transferase (GGT) and lactate dehydrogenase (LDH) activity) was performed using an Architect C4000 analyzer in conformity with the original working protocols of the manufacturer (Abbott Laboratories, IL, USA). Complete blood count determination was achieved on a Mindray BC6200 (Mindray, Shenzhen, China). Derived blood count parameters, like neutrophil-to-lymphocyte ratio (NLR), systemic inflammatory response index (SIRI), and the aggregate index of systemic inflammation (AISI), were calculated as previously described [30]. Plasma fibrinogen was measured using a Sysmex CA-1500 (Sysmex Corporation, Kobe, Japan), while N-terminal pro-B-type natriuretic peptide (NT-proBNP) concentrations were determined on Elecsys 2010 immunology analyzer by means of electrochemiluminescence (Roche, Rotkreuz, Switzerland). The quantitative determination of the total 25-hydroxyvitamin was performed using a competitive electrochemiluminescence protein binding assay on a Mindray CL-900i Chemiluminescence Immunoassay Analyzer (Mindray Bio-Medical Electronics Co., Ltd., Shenzhen, China).

### 2.5. Statistical Analysis

The Shapiro–Wilk and Kolmogorov–Smirnov tests were employed to determine data distribution. For variables exhibiting a normal distribution, paired *t*-tests and Pearson’s correlation analyses were conducted. In cases where data did not follow a Gaussian distribution, the Mann–Whitney U test and Spearman’s rank correlation were applied. Categorical variables were expressed as absolute and relative frequencies, and 2 × 2 contingency tables were evaluated using the chi-square (χ^2^) test. Principal component analysis (PCA) was used for the dimensionality reduction in data by creating new variables called principal components (PCs), in order to highlight the structure (clusters) in a large set of variables. These were the following: albumin, uric acid, ferritin, thyroid-stimulating hormone levels (TSH), GGT activity, NT-proBNP, 25-hydroxyvitamin D, IL-6, MIF-1, hemoglobin, erythrocyte sedimentation rate (ESR), mean platelet volume (MPV), NLR, absolute monocyte count, and SIRI, BMI, LVEF, TAPSE, six-minute walk distance, and ankle-brachial index (ABI), all as untransformed values

Statistical significance was defined as *p* < 0.05. Data curation and analysis were performed using Microsoft Excel 2016 (Microsoft Corporation, Redmond, WA, USA) and GraphPad Prism version 9.5.0 (GraphPad Software LLC, San Diego, CA, USA).

## 3. Results

### 3.1. Study Group Characteristics

**Figure 1:**
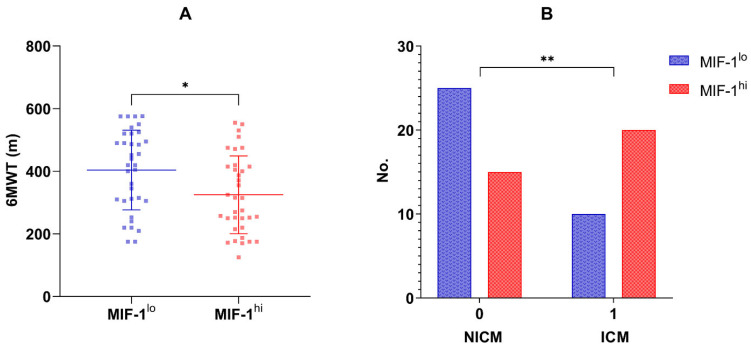
(**A**). Six-minute walk distance (m) in the MIF-1^lo^ vs. MIF-1^hi^ subgroups; means and standard deviation values are also illustrated; * *p*-value = 0.010. (**B**). Distribution of non-ischemic and ischemic cardiomyopathy in the MIF-1^lo^ and MIF-1^hi^ subgroups; ** *p*-value = 0.029.

Seventy patients (51 men, 72.85%) were enrolled in the current study. The mean age at recruitment was 66 ± 11 years. The median concentration of MIF-1 in the overall population was 520 pg/mL (IQR 317.26–1219.38 pg/mL). Table 1, Table 2 and Table 3 summarize the main characteristics of the overall study group, and compare the two subgroups, MIF-1^lo^ vs. MIF-1^hi^, which were divided based on the median MIF-1 concentration. Patients with HFrEF had higher circulating MIF-1 levels (661.64, IQR 335.67–1575.26 pg/mL) compared to those with HFmrEF (393.06, IQR 301.11–740.09 pg/mL), but the difference did not reach statistical significance. Also, when considering LVEF as a continuous variable, a statistically significant inverse relationship was reported regarding MIF-1 (r = −0.33, *p* = 0.005). LVGLS proved to be an even stronger correlating echocardiographic marker (r = 0.41, *p* = 0.0004). Moreover, ischemic cardiomyopathy (ICM) was more frequent in the MIF-1^hi^ group (20 vs. 10 patients, *p* = 0.029; Figure 1B).

Echocardiographic markers of right ventricular (RV) systolic function were also assessed, and a statistically significant, negative correlation was found between MIF-1 and TAPSE (r = −0.37, *p* = 0.001) in our patient cohort (*n* = 70). Also, TAPSE values were rather in the normal range in the MIF-1^lo^ subgroup (22.0 mm, IQR 20.0–24.0 mm vs. 17.0 mm, IQR 15.0–22.0 mm; *p* = 0.002). Moreover, MIF-1 > 520 pg/mL proved to be a significant determinant for low TAPSE (*p* = 0.041) among other parameters—the upper tertiles of GGT (*p* = 0.015) and NT-proBNP (*p* = 0.005)—in Model 2, even after adjusting for various confounders, such as gender, the habit of smoking, comorbidities (chronic obstructive pulmonary disease (COPD), hypertension, coronary artery disease (CAD)) and laboratory parameters (absolute monocyte count, HDL-cholesterol and TSH levels). No relationship was reported between MIF-1 and sPAP (r = 0.11, *p* = 0.351). However, MIF-1 showed significant associations with TAPSE/sPAP (r = −0.24, *p* < 0.0001) in the overall patient population (*n* = 70). Also, patients with TAPSE/sPAP < 0.40 mm/mmHg had significantly higher concentrations of MIF-1 (*p* = 0.009).

Furthermore, patients with higher levels of circulating MIF-1 showed longer hospital stays (r = 0.51, *p* ≤ 0.0001) and greater BMI (r = 0.27, *p* = 0.023). Individuals in the MIF-1^hi^ group had longer-lasting heart disease (8 vs. 12 years, *p* = 0.028), lower levels of serum albumin (42.7, IQR 38.1–46.2 g/L vs. 45.2, IQR 42.8–46.8 g/L, *p* = 0.035), and worse kidney function (eGFR 64.9 ± 3.3 vs. 77.0 ± 3.7 mL/min/m^2^, *p* = 0.019). Regarding MIF-1, we did not observe statistically significant correlations with the white blood cell (WBC) count (r = −0.13, *p* = 0.276) and CRP concentrations (r = 0.09, *p* = 0.483). When considering levels of circulating IL-6, statistically significant differences were found between the two MIF groups, high versus low (4.9, IQR 2.7–10.8 pg/mL vs. 2.9, IQR 1.1–6.0 pg/mL, *p* = 0.015).

In terms of congestion, MIF-1 showed significant associations with the presence of peripheral edema (*p* = 0.007), but none was found with self-reported dyspnea (*p* = 0.307) and New York Heart Association (NYHA) class (*p* = 0.486). Also, no relationship was reported with NT-proBNP (r = 0.14, *p* = 0.263). However, the six-minute walk distance was greater in individuals in the MIF-1^lo^ group when compared to those in the MIF-1^hi^ group (404.0 ± 127.4 m vs. 324.8 ± 124.1 m, *p* = 0.010; Figure 1A).

### 3.2. Principal Component Analysis on the Overall Study Group

PCA was used to define clinical, laboratory and ultrasound parameters that accounted for most of the variance in our data sets. The initial model included a total of 20 variables and was used as a component selection method for a parallel analysis. These were continuous biochemical and immunological variables (among which albumin, uric acid, ferritin, TSH, GGT, NT-proBNP, 25-hydroxyvitamin D, IL-6, and MIF-1), other laboratory parameters potentially affecting myocardial function (hemoglobin, ESR, MPV, NLR, absolute monocyte count, and SIRI), BMI, and 4 cardiovascular functional measures: LVEF, TAPSE, six-minute walk distance, and ABI. Twenty PCs were defined, of which the first ten accounted for more than 80% of the total variance in the data, while eight PCs possessed an eigenvalue above 1. PC1, PC2, and PC3 accounted for 17.8%, 13.5%, and 10.5% of the data variation, being included in further analysis.

**Figure 2:**
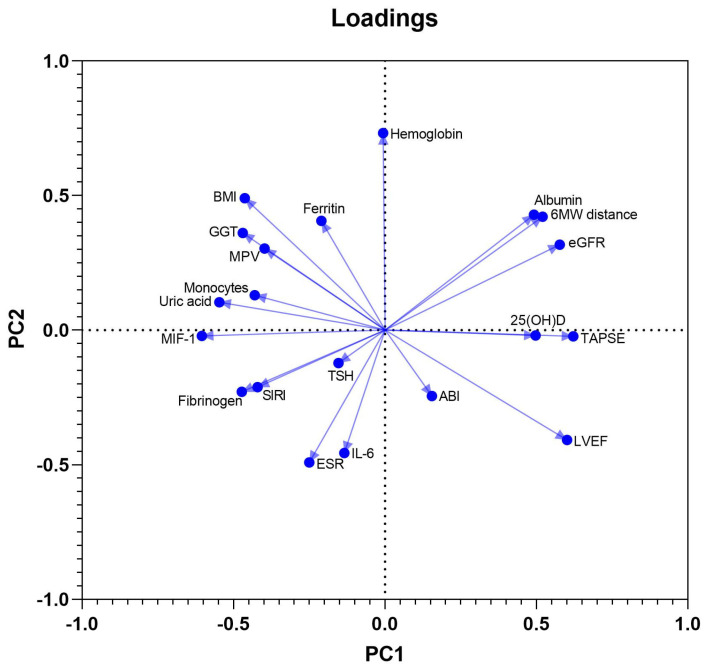
Loadings of PC1 vs. PC2.

MIF-1, SIRI, and uric acid had negative coefficients in component 1, in contrast to the positive coefficients of LVEF, TAPSE, and 25-hydroxyvitamin D, reflecting probably the negative effects of systemic inflammation and higher oxidative stress on LV and RV systolic function. The PC2 had positive associations with BMI, ferritin, and hemoglobin, and negative associations with NT-proBNP, NLR, and ESR. The third component, PC3, showed positive coefficients of MIF-1 and IL-6, but, in contrast, negative coefficients of SIRI, NLR, and monocyte count, most likely pointing towards a dissociation of the monocyte migration inhibitor, and intimate quantitative variations of circulating inflammatory cells. The loadings of PC1 vs. PC2 are shown in Figure 2.

### 3.3. Multiple Logistic Regression Analysis

**Figure 3:**
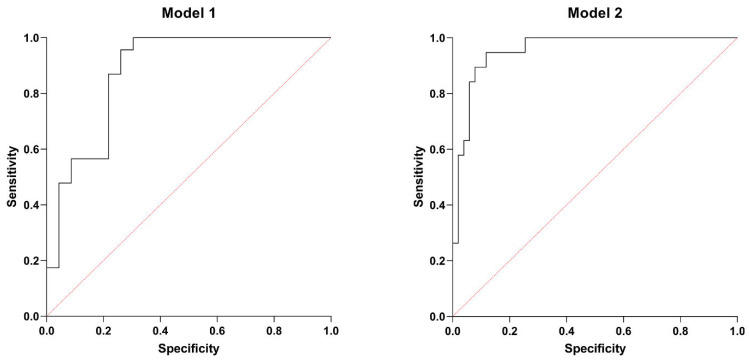
ROC curves: Model 1. AUC of 0.877 (0.773–0.980), a positive and negative predictive value of 78.26%. Model 2. AUC of 0.955 (0.911–1.000), a positive predictive value of 80%, and a negative predictive value of 94%.

Based on the PCA (Table 4) and further correlation analysis (Table 5), two multiple logistic regression models were designed to predict low LVEF and right ventricular systolic dysfunction. The first model (Model 1) predicted the lowest tertile of LVEF with *p* < 0.001 and an area under (AUC) the receiver operating characteristic curve (ROC) of 0.877 (0.773–0.980), a positive and negative predictive value of 78.26%. SIRI, higher serum MIF-1, and high GGT activity were significant predictors of lower (tertile 1) LVEF. Table 6 shows the characteristics of Model 1, while Figure 3 illustrates the ROC curve of this model.

Model 2 predicted low TAPSE with *p* < 0.001, AUC of ROC of 0.955 (0.911–1.000), a positive predictive value of 80%, and a negative predictive value of 94%. GGT, NT-proBNP, and MIF-1 proved to be significant determinants, even after adjusting for possible confounders, such as gender, the habit of smoking, comorbidities (COPD, hypertension, CAD), and laboratory parameters (absolute monocyte count, HDL-cholesterol, TSH levels). Table 7 summarizes the main characteristics of Model 2, while Figure 3 displays the ROC curve of this model.

## 4. Discussion

The monocyte-macrophage system plays a crucial role in cardiovascular homeostasis. Both pro-inflammatory (M1, CCR2+) and anti-inflammatory (M2, CCR2−) cardiac macrophages were identified within ischemic and non-ischemic failing hearts [31]. Nonetheless, the functional equilibrium and the phenotype switch between these two are of particular interest, especially in myocardial I/R injury. MIF proved to be a mediator and marker of the extent of myocardial necrosis, facilitating both atherogenesis and atherosclerosis [32,33]. MIF enhances myeloid and T cell infiltration, thus promoting local inflammation and atherosclerotic plaque destabilization mainly by CXCR2/4 signaling [13]. Moreover, MIF was previously associated with the presence of CAD [32,34]. It is important to note that ICM was more frequent in the MIF-1^hi^ group. Also, MIF and hypoxia-inducible factor 1α expression were increased in myocardial samples of patients with ICM [35]. Mueller et al. identified positive correlations between the expression rate of MIF in cardiomyocytes and the degree of local fibrosis in non-ischemic cardiomyopathy (NICM) (LVEF < 55%) as well [36]. Adverse ventricular remodeling is a key contributor to HF and is associated with poor prognosis [37]. The rs755622 G/C single nucleotide polymorphism of the MIF gene could represent a genetic risk factor for HF, especially in HFrEF, driving disease progression [38]. Furthermore, circulating MIF-1 was strongly associated with both LVEF (*p* = 0.005) and LVGLS (*p* = 0.0004) in the overall patient population. Although patients with HFrEF had higher levels of MIF-1, the difference was not significant (*p* = 0.116). Multiple logistic regression analysis was also performed in order to predict low LVEF. Elevated concentrations of MIF-1, high GGT activity, and increased SIRI were all significant indicators of LV systolic dysfunction.

RV function was also assessed by means of echocardiography. TAPSE showed significant associations with MIF-1 (*p* = 0.001); moreover, patients in the MIF-1^lo^ subgroup had better RV function (*p* = 0.002). Also, MIF-1 > 520 pg/mL proved to be a significant determinant for low TAPSE in the second multiple logistic regression analysis (*p* = 0.041). However, Luedike et al. did not report significant differences regarding values of TAPSE according to extreme tertiles (T1 vs. T3) of MIF (*p* = 0.96) [16]. TAPSE to sPAP ratio is a non-invasive echocardiographic marker of RV-PA coupling of prognostic significance. TAPSE/sPAP < 0.40 mm/mmHg assessed early during hospitalization for acute HF was predictive of in-hospital major adverse cardiovascular events (MACEs, defined as the composite of cardiogenic shock, all-cause mortality, and resuscitated cardiac arrest) [27]. MIF-1 showed significant associations with TAPSE/sPAP (*p* < 0.0001). Also, patients with TAPSE/sPAP < 0.40 mm/mmHg had significantly higher concentrations of MIF-1 (*p* = 0.009).

Pulmonary hypertension (PH) is a common condition in HF and is linked to increased mortality. Regardless of LVEF, PH is observed in the majority of HF patients, with prevalence reaching up to 83%. The underlying pathophysiology of PH in HF is primarily driven by the retrograde transmission of elevated LV filling pressures into the pulmonary circulation, leading to post-capillary PH. Secondary changes may lead to pulmonary arterial remodeling, further aggravating PH [39]. Perivascular inflammation—the overexpression of adhesion molecules and upstream mediators (ILs, chemokines)—induces functional and structural maladaptive changes within the pulmonary vessels [40]. MIF proved to be a key contributor to the development of PH [41]. Moreover, fluid overload may trigger MIF secretion. Luedike et al. also examined the prognostic significance of MIF in both HFrEF and HF with preserved EF (HFpEF) [16]. Contrary to their results, we did not observe statistically significant correlations with the WBC count, CRP concentrations, sPAP, and NYHA functional class. In HFpEF, a close relationship was reported between MIF, sPAP, and natriuretic peptides (both BNP and NT-proBNP). Also, high circulating MIF levels were associated with all-cause mortality at 180 days [42]. The lack of relationship between MIF-1 and sPAP/NT-proBNP/NYHA class could be explained by the hemodynamic status of our patients, who were included in the study in the absence of clinical congestion, showing only mildly elevated LV filling pressures (E/e’ = 10.1). It is important to note that participants were recruited either from ambulatory care or were inpatients hospitalized for worsening HF, and evaluated before discharge after intensification and dose adjustment of diuretic therapy. That being said, NT-proBNP levels were measured after decongestion (when levels decreased significantly), and the NYHA class was also updated based on the patient’s clinical status. However, the presence of peripheral edema was associated with elevated circulating MIF-1. Also, the same interaction was reported by Luedike et al. in HF regardless of LVEF [16]. These findings further support the role of MIF in HF regarding fluid overload (congestion). Also, MIF is abundantly secreted in end-stage chronic kidney disease (CKD) and is filtered during hemodialysis. MIF concentrations were observed to return to baseline shortly after the end of the hemodialysis session, likely due to its release from intracellular storage vesicles [43]. Also, our patients with high MIF-1 concentrations had worse kidney function.

Fatty tissue has been proven to be active both hormonally and immunologically. Visceral fat contains elements of the monocyte-macrophage system that abundantly secrete TNF-α [44,45]. MIF-1 showed a statistically significant association with BMI. Physical exercise reduced the number of M1-type (inflammatory) macrophages, derived from monocytes, in the adipose tissue of obese mice, and promoted the polarization of macrophages toward the reparative M2 phenotype [46]. Caloric restriction to reduce body fat led to decreased concentrations of circulating TNF-α, IL-6, leptin, and plasminogen activator inhibitor 1 in a group of obese women [47]. In a Greek population of 3042 individuals aged 18–89, a BMI > 29.9 kg/m^2^ was associated with higher levels of CRP, TNF-α, IL-6, and WBC count [48]. MIF stimulates the release of a significant number of pro-inflammatory cytokines, including TNF-α, IL-1, IL-6, IL-8, and IL-12. In HF, elevated circulating IL-6 concentrations have been associated with increased levels of NT-proBNP and renin [49]. In our patient population, a positive correlation was identified between MIF-1 and IL-6. IL-6 is a pro-inflammatory cytokine associated with abnormal LV remodeling and dysfunction [50]. Also, the PCA was strongly indicative of the close relationship between markers of systemic inflammation and LV/RV systolic function. It is important to note that regular physical exercise has antioxidant and anti-inflammatory effects [51].

## 5. Conclusions

Circulating MIF-1 can be detected in patients with HFrEF and HFmrEF and correlates with inflammatory markers and clinical and paraclinical parameters of disease severity. The numerous interactions of MIF-1 and IL-6 with established biomarkers in HF highlight the active involvement of systemic inflammation and the monocyte-macrophage system in the complex pathophysiology of HF, influencing congestion and fluid overload.

Further in vivo and in vitro studies are necessary to draw robust conclusions regarding the role played by MIF-1 in HF. The pharmacological antagonization of MIF could provide potential benefits in reducing cardiovascular morbidity and mortality in HF.

### Limitations

The main limitation is the relatively small number of participants. Also, it is important to note the observational nature of the study and the lack of a control group consisting of healthy individuals. Therefore, a more appropriate research design and larger studies are necessary to provide more accurate and reliable results.

## Figures and Tables

**Table 1:** Demographic, lifestyle factors, and clinical data of the overall study group, MIF-1^lo^, and MIF-1^hi^ subgroups.

Characteristics	All Patients, *n* = 70	MIF-1^lo^, *n* = 35	MIF-1^hi^, *n* = 35	*p*
Inpatient/outpatient, no. (%)	39 (55.7)/31 (44.3)	23 (65.7)/12 (34.3)	8 (22.8)/27 (77.2)	0.0006
Male/female, no. (%)	51 (72.9)/19 (27.1)	25 (71.4)/10 (28.6)	26 (74.3)/9 (25.7)	1.000
Age, mean ± SD (min; max), years	66.0 ± 10.9 (40.0; 88.0)	64.6 ± 10.8 (41.0; 81.0)	67.3 ± 11.0 (40.0; 88.0)	0.309
BMI, mean ± SD, kg/m^2^	28.6 ± 5.5	27.2 ± 4.5	29.9 ± 6.1	0.041
History of heart disease, median (IQR), years	8.0 (4.0–15.0)	8.0 (4–10)	12.0 (4–20)	0.028
HF, median (IQR), years	2.0 (1.0–8.0)	2 (1–7)	3 (1–10)	0.423
NYHA class I/II/III, no. (%)	7 (10.0)/43 (61.4)/20 (28.6)	5 (14.3)/22 (62.8)/8 (22.9)	2 (5.7)/21 (60)/12 (34.3)	0.348
Dyspnea (yes/no)	60 (85.7)/10 (14.3)	27 (77.1)/8 (22.9)	33 (94.3)/2 (5.7)	0.084
Edema (yes/no)	18 (25.7)/52 (74.3)	5 (14.3)/30 (85.7)	13 (37.1)/22 (62.9)	0.053
6MWT, mean ± SD, m	365.5 ± 131.1	404.0 ± 127.4	324.8 ± 124.1	0.010
MLHFQ score, median (IQR)	20.0 (10.0–40.0)	16.0 (8–31)	23.0 (12–48)	0.103
Smoking, yes/no	17 (24.3)/53 (75.7)	12 (34.3)/23 (65.7)	5 (14.3)/30 (85.7)	0.093
Hypertension, yes/no	38 (54.3)/32 (45.7)	19 (50)/19 (50)	19 (50)/19 (50)	1.000
ICM/NICM	30 (42.9)/40 (57.1)	10 (28.6)/25 (71.4)	20 (57.1)/15 (42.9)	0.029
Mitral valve regurgitation medium/severe, yes/no	28 (40.0)/42 (60.0)	14 (40)/21 (60)	14 (40)/21 (60)	1.000
Aortic stenosis medium/severe, yes/no	9 (12.9)/61 (87.1)	4 (11.4)/31 (88.8)	5 (14.3)/30 (85.7)	1.000
Tricuspid regurgitation medium/severe, no. (%)	20 (28.6)/50 (71.4)	9 (25.7)/26 (74.3)	11 (31.4)/24 (68.6)	0.792
AF/AFL, yes/no	32 (45.7)/38 (54.3)	14 (40)/21 (60)	18 (51.4)/17 (48.6)	0.472
Type 2 DM, yes/no	21 (30.0)/49 (70.0)	10 (28.6)/25 (71.4)	11 (31.4)/24 (68.6)	1.000
Symptomatic PAD, yes/no	9 (12.9)/61 (77.1)	5 (14.3)/30 (85.7)	4 (11.4)/31 (88.8)	1.000
COPD, yes/no	12 (17.1)/58 (82.9)	6 (20.7)/29 (79.3)	6 (20.7)/29 (79.3)	1.000

SD, standard deviation; BMI, body mass index; IQR, interquartile range; HF, heart failure; NYHA, New York Heart Association; 6MWT, six-minute walk distance; MLHFQ, Minnesota Living with Heart Failure Questionnaire; ICM, ischemic cardiomyopathy; NICM, non-ischemic cardiomyopathy; AF, atrial fibrillation; AFL, atrial flutter; DM, diabetes mellitus; PAD, peripheral arterial disease; COPD, chronic obstructive pulmonary disease. *p*-values indicate the statistical significance of differences observed between the MIF-1^hi^ and MIF-1^lo^ subgroups.

**Table 2:** Echocardiography findings of the overall study group, MIF-1^lo^ and MIF-1^hi^ subgroups.

Characteristics	All Patients, *n* = 70	MIF-1^lo^, *n* = 35	MIF-1^hi^, *n* = 35	*p*
LUS profile 0/1, no. (%)	53 (75.7)/17 (24.3)	29 (82.9)/6 (17.1)	24 (68.6)/11 (31.4)	0.265
LAVI, median (IQR), mL/m^2^	47.0 (37.1–65.1)	45.4 (33.8–55.1)	47.8 (38.9–79.4)	0.120
E/e’, median (IQR)	10.1 (8.1–12.8)	9.7 (7.9–11.8)	11.2 (8.1–13.3)	<0.0001
LVEF, mean ± SD, %	36.0 ± 8.8	38.3 ± 8.4	33.7 ± 8.8	<0.0001
LVGLS, mean ± SD, %	−11 ± 3.5	−12.4 ± 3.3	−9.6 ± 3.2	<0.0001
SVI, mean ± SD, mL/m^2^	32.8 ± 11.1	35.1 ± 11.4	30.5 ± 1.8	<0.0001
TAPSE, median (IQR), mm	21.0 (16.0–23.0)	22.0 (20.0–24.0)	17.0 (15.0–22.0)	0.002
sPAP, mean ± SD, mmHg	33.8 ± 12.4	32.2 ± 10.1	35.4 ± 14.4	0.285
TAPSE/sPAP, mean ± SD, mm/mmHg	0.7 ± 0.3	0.7 ± 0.3	0.6 ± 0.3	0.094

LUS, lung ultrasound; LAVI, left atrial volume indexed; IQR, interquartile range; LVEF, left ventricular ejection fraction; SD, standard deviation; LVGLS, left ventricular global longitudinal strain; SVI, stroke volume indexed; TAPSE, tricuspid annular plane systolic excursion; sPAP, systolic pulmonary artery pressure. *p*-values indicate the statistical significance of differences observed between the MIF-1^hi^ and MIF-1^lo^ subgroups.

**Table 3:** Laboratory variables of the overall study population, MIF-1^lo^ and MIF-1^hi^ subgroups.

Characteristics	All Patients, *n* = 70	MIF-1^lo^, *n* = 35	MIF-1^hi^, *n* = 35	*p*
WBC, mean ± SD, ×1000/µL	7.3 ± 2.1	7.6 ± 1.9	7.0 ± 2.2	0.205
Monocyte count, mean ± SD, ×1000/µL	0.6 ± 0.0	0.56 ± 0.03	0.57 ± 0.03	0.648
NLR, mean ± SD	2.7 ± 0.1	2.83 ± 0.27	2.55 ± 0.21	0.879
SIRI, mean ± SD	1.6 ± 0.1	1.64 ± 0.19	1.49 ± 0.15	0.815
AISI, mean ± SD	389.2 ± 35.3	432.7 ± 54.3	345.6 ± 44.7	0.256
Platelet count, mean ± SD, ×1000/µL	238.4 ± 67.0	256.3 ± 10.7	220.4 ± 11.2	0.013
Hemoglobin, mean ± SD, g/dL	14.5 ± 2.0	14.5 ± 0.3	14.6 ± 0.3	0.851
Ferritin, median (IQR), ng/dL	119.7 (72.5–202.2)	157.0 (78–219)	171.9 (66–183)	0.447
Iron, median (IQR), μg/dL	81.5 (56.0–114.0)	82.0 (59–116)	81.0 (55–114)	0.962
Cholesterol, mean ± SD, mg/dL	171.5 ± 46.8	177.2 ± 7.5	165.7 ± 8.2	0.223
Triglycerides, median (IQR), mg/dL	111.5 (83.0–138.0)	127.5 ± 14.2	127.9 ± 11.2	1.000
CRP, median (IQR), mg/dL	0.3 (0.1–0.8)	0.4 (0.1–0.7)	0.4 (0.1–1.2)	0.256
Fibrinogen, mean ± SD, mg/dL	400.0 ± 127.4	383.7 ± 21.2	416.3 ± 21.8	0.337
Albumin, median (IQR), g/L	44.6 (40.1–46.3)	45.2 (42.8–46.8)	42.7 (38.1–46.2)	0.035
eGFR, mean ± SD, mL/min/m^2^	71.0 ± 21.5	77.0 ± 3.7	64.9 ± 3.3	0.019
Uric acid, mean ± SD, mg/dL	6.8 ± 2.4	6.3 ± 0.4	6.8 ± 0.4	0.074
NT-proBNP, median (IQR), pg/mL	967.3 (547.5–1890.5)	891.8 (366.6–1576.4)	1274.1 (607.8–3020.8)	0.143
IL-6, median (IQR), pg/mL	4.5 (0.2–8.8)	2.9 (1.1–6.0)	4.9 (2.7–10.8)	0.015

WBC, white blood cells; SD, standard deviation; NLR, neutrophil-to-lymphocyte ratio; SIRI, systemic inflammatory response index; AISI, aggregate index of systemic inflammation; IQR, interquartile range; CRP, C-reactive protein; eGFR, estimated glomerular filtration rate; NT-proBNP, N-terminal pro-B-type natriuretic peptide; IL-6, interleukin 6. *p*-values indicate the statistical significance of differences observed between the MIF-1^hi^ and MIF-1^lo^ subgroups.

**Table 4:** Principal component analysis defining PCs 1–10, their variance, and eigenvalues.

PC Summary	PC1	PC2	PC3	PC4	PC5	PC6	PC7	PC8	PC9	PC10
Eigenvalue	3.56	2.69	2.09	1.59	1.38	1.21	1.09	1.02	0.83	0.78
Proportion of variance	17.79%	13.48%	10.45%	7.99%	6.94%	6.10%	5.45%	5.10%	4.19%	3.95%
Cumulative proportion of variance	17.79%	31.28%	41.73%	49.72%	56.66%	62.75%	68.20%	73.30%	77.50%	81.44%

**Table 5:** Clinical and laboratory data correlations with MIF-1 in the overall population.

Characteristics	All Patients, *n* = 70		Characteristics	All Patients, *n* = 70	
	r	*p*		r	*p*
Hospitalization, days	0.51	<0.0001	WBC, ×1000/µL	−0.13	0.276
Age, years	0.03	0.811	Neutrophils, ×1000/µL	−0.16	0.175
BMI kg/m^2^	0.27	0.023	Lymphocytes, ×1000/µL	0.03	0.810
Heart disease, years	0.15	0.212	NLR	−0.07	0.570
History of HF, years	0.06	0.653	Monocytes, ×1000/µL	0.06	0.600
SBP, mmHg	−0.28	0.021	Platelets, ×1000/µL	−0.22	0.070
DBP, mmHg	−0.22	0.064	MPV, Fl	0.11	0.357
HR, bpm	0.10	0.415	PDW, %	0.15	0.228
ABI	−0.03	0.819	Hemoglobin, g/dL	0.06	0.597
6MWT, m	−0.23	0.061	Uric acid, mg/dL	0.26	0.031
LAVI, mL/m^2^	0.15	0.210	Albumin, g/L	−0.23	0.061
E/e’	0.15	0.230	Cholesterol, mg/dL	−0.18	0.133
LVEDVI, mL/m^2^	0.07	0.565	HDL-cholesterol, mg/dL	−0.28	0.020
LVESVI, mL/m^2^	0.17	0.169	Glycemia, mg/dL	0.06	0.616
LVEF, %	−0.33	0.005	Triglycerides, mg/dL	0.08	0.496
LVGLS, %	0.41	0.0004	GGT, UI/L	0.19	0.123
SVI, mL/m^2^	−0.17	0.157	LDH, UI/L	0.24	0.049
TAPSE, mm	−0.37	0.001	TSH, µU/mL	0.30	0.012
sPAP, mmHg	0.11	0.351	Iron, µg/dL	<0.01	0.999
TAPSE/sPAP, mm/mmHg	−0.24	<0.0001	Ferritin, ng/dL	−0.04	0.758
NT-proBNP, pg/mL	0.14	0.263	Fibrinogen, mg/dL	0.16	0.186
25(OH)D, ng/mL	−0.27	0.022	CRP, mg/dL	0.09	0.483
eGFR, mL/min/1.73 m^2^	−0.30	0.011	IL-6, pg/mL	0.25	0.049

BMI, body mass index; HF, heart failure; SBP, systolic blood pressure; DBP, diastolic blood pressure; HR, heart rate; bpm, beats per minute; ABI, ankle-brachial index; 6MWT, six-minute walk distance; LAVI, left atrial volume index; LVEDVI, left ventricular end-diastolic volume index; LVESVI, left ventricular end-systolic volume index; LVEF, left ventricular ejection fraction; LVGLS, left ventricular global longitudinal strain; SVI, stroke volume index; TAPSE, tricuspid annular plane systolic excursion; sPAP, systolic pulmonary artery pressure; NT-proBNP, N-terminal pro B-type natriuretic peptide; 25(OH)D, 25-hydroxyvitamin D; eGFR, estimated glomerular filtration rate; WBC, white blood cells; NLR, neutrophil-to-lymphocyte ratio; MPV, mean platelet volume; PDW, platelet distribution width; HDL, high-density lipoprotein; GGT, gamma-glutamyl transferase; LDH, lactate dehydrogenase; TSH, thyroid-stimulating hormone; CRP, C-reactive protein; IL-6, interleukin 6.

**Table 6:** Model 1—summary of the multiple logistic regression analysis of the factors predicting low LVEF in the overall patient cohort.

Odds Ratios	Variable	Estimate	95% CI (Profile Likelihood)	*p*
β0	Intercept	7387	75.16 to 4,379,945	0.001
β1	SIRI	0.382	0.145 to 0.839	0.028
β2	Uric acid (T3:T1)	0.521	0.177 to 1.409	0.206
β3	GGT (T3:T1)	0.209	0.056 to 0.590	0.007
β4	MIF-1 (U:L)	0.169	0.028 to 0.793	0.033

T1—tertile 1, T3—tertile 3, U—upper 50%, L—lower 50%. SIRI, systemic inflammatory response index; GGT, gamma-glutamyl transferase; MIF-1, macrophage migration inhibitory factor 1.

**Table 7:** Model 2—summary of the multiple logistic regression analysis of the factors predicting low TAPSE in the overall patient cohort.

Odds Ratios	Variable	Estimate	95% CI (Profile Likelihood)	*p*
β0	Intercept	1.68 × 10^−8^	3.036 × 10^−15^ to 0.0002823	0.004
β1	Gender (male:female)	0.87	0.041 to 21.34	0.926
β2	Smoking (yes:no)	0.352	0.027 to 3.964	0.392
β3	Hypertension (yes:no)	0.339	0.036 to 2.446	0.296
β4	CAD (yes:no)	0.318	0.025 to 2.699	0.319
β5	Type 2 DM (yes:no)	1.666	0.215 to 15.08	0.625
β6	COPD (yes:no)	2.753	0.250 to 37.01	0.412
β7	Monocyte count	10.700	0.045 to 2939	0.385
β8	HDL-cholesterol (T3:T1)	0.749	0.222 to 2.360	0.619
β9	GGT (T3:T1)	7.896	1.903 to 61.73	0.015
β10	NT-proBNP (T3:T1)	9.924	2.558 to 70.35	0.005
β11	TSH (T3:T1)	3.150	0.935 to 14.12	0.085
β12	MIF-1 (U:L)	17.790	1.660 to 540.2	0.041

T1—tertile 1, T3—tertile 3, U—upper 50%, L—lower 50%. CAD, coronary artery disease; DM, diabetes mellitus; COPD, chronic obstructive pulmonary disease; HDL, high-density lipoprotein; GGT, gamma-glutamyl transferase; N-terminal pro-B-type natriuretic peptide; TSH, thyroid-stimulating hormone; MIF-1, macrophage migration inhibitory factor 1.

## Data Availability

Data spreadsheets are available as “Macrophage migration inhibitory factor 1 is a marker of both left and right ventricular systolic dysfunction in heart failure with reduced ejection fraction”. DOI 10.6084/m9.figshare.28633052 (accessed on 30 March 2025).

## Abbreviations

The following abbreviations are used in this manuscript:

25(OH)D	25-hydroxyvitamin D
6MWT	Six-minute walk test distance
ABI	Ankle-brachial index
AF	Atrial fibrillation
AFL	Atrial flutter
AISI	Aggregate index of systemic inflammation
AUC	Area under the curve
BMI	Body mass index
CAD	Coronary artery disease
CKD	Chronic kidney disease
COPD	Chronic obstructive pulmonary disease
CRP	C-reactive protein
DBP	Diastolic blood pressure
D-DT	D-dopachrome tautomerase
DM	Diabetes mellitus
eGFR	Estimated glomerular filtration rate
ESR	Erythrocyte sedimentation rate
GGT	Gamma-glutamyl transferase
HDL	High-density lipoprotein
HF	Heart failure
HFmrEF	Heart failure with mildly reduced ejection fraction
HFpEF	Heart failure with preserved ejection fraction
HFrEF	Heart failure with reduced ejection fraction
HR	Heart rate
I/R	Ischemia/reperfusion
ICM	Ischemic cardiomyopathy
IL	Interleukin
LAVI	Left atrial volume indexed
LDH	Lactate dehydrogenase
LDL	Low-density lipoprotein
LUS	Lung ultrasound
LVEDVI	Left ventricular end-diastolic volume index
LVEF	Left ventricular ejection fraction
LVESVI	Left ventricular end-systolic volume index
LVGLS	Left ventricular global longitudinal strain
MACEs	Major adverse cardiovascular events
MIF	Macrophage migration inhibitory factor
MLHFQ	Minnesota Living with Heart Failure Questionnaire
MPV	Mean platelet volume
mRNA	Messenger ribonucleic acid
NICM	Non-ischemic cardiomyopathy
NLR	Neutrophil-to-lymphocyte ratio
NT-proBNP	N-terminal pro-B-type natriuretic peptide
NYHA	New York Heart Association
PAD	Peripheral arterial disease
PCA	Principal component analysis
PDW	Platelet distribution width
PH	Pulmonary hypertension
ROC	Receiver operating curve
RV	Right ventricle/ventricular
RV-PA	Right ventricular-pulmonary artery
SBP	Systolic blood pressure
SIRI	Systemic inflammatory response index
sPAP	Systolic pulmonary artery pressure
SVI	Stroke volume indexed
TAPSE	Tricuspid annular plane systolic excursion
TNF-α	Tumor necrosis factor α
TSH	Thyroid-stimulating hormone
WBC	White blood cells
